# Association of Simulated Patient Race/Ethnicity With Scheduling of Primary Care Appointments

**DOI:** 10.1001/jamanetworkopen.2019.20010

**Published:** 2020-01-29

**Authors:** Janna M. Wisniewski, Brigham Walker

**Affiliations:** 1 School of Public Health and Tropical Medicine, Department of Health Policy and Management, Tulane University, New Orleans, Louisiana; 2 Department of Economics, Tulane University, New Orleans, Louisiana

## Abstract

**Importance:**

Racial and ethnic disparities in access to health care may result from discrimination.

**Objectives:**

To identify differences in the rates at which patients belonging to racial/ethnic minority groups are offered primary care appointments and the number of days they wait for their primary care appointment and to understand the mechanisms by which discrimination occurs.

**Design, Setting, and Participants:**

This cross-sectional study used 7 simulated black, Hispanic, and white patient callers to request appointments from 804 randomized primary care offices in 2 urban centers in Texas from November 2017 to February 2018. Data analysis was conducted between February and December 2018.

**Exposures:**

Research assistants called randomly assigned offices to schedule an appointment, supplying the same basic information. Race and ethnicity were signaled through callers’ names and voices.

**Main Outcomes and Measures:**

Appointment offer rates, days to appointment, and questions asked during the call.

**Results:**

Of the 7 callers (age range, 18-29 years), 2 (28.6%) self-identified as non-Hispanic black, 3 (42.9%) self-identified as non-Hispanic white, and 2 (28.6%) self-identified as Hispanic. Of the 804 calls they made, 299 (37.2%) were from simulated white callers, 215 (26.7%) were from simulated black callers, and 290 (36.1%) were from simulated Hispanic callers. Overall, 582 callers (72.4%) were offered appointments. In unadjusted models, black and Hispanic callers were more likely to be offered an appointment than white callers (black callers, 32.2 [95% CI, 25.1-39.3] percentage points more likely; *P* < .001; Hispanic callers, 21.1 [95% CI, 13.7-28.5] percentage points more likely; *P* < .001). However, after adjusting for whether insurance status was revealed, this statistical significance was lost. In adjusted models, black callers were 44.0 (95% CI, 36.2-51.8) percentage points more likely to be asked about their insurance status than white callers (*P* < .001), and Hispanic callers were 25.3 (95% CI, 17.1-33.5) percentage points more likely to be asked about their insurance status (*P* < .001) than white callers. Black and Hispanic callers received appointments further in the future than white callers (black callers: marginal effect estimate, 3.650; 95% CI, 0.579 to 6.721; *P* = .08; Hispanic callers: marginal effect estimate, 2.644; 95% CI, −0.496 to 5.784; *P* = .02).

**Conclusions and Relevance:**

In this study, black and Hispanic patients were more likely to be offered an appointment, but they were asked more frequently about their insurance status than white callers. Black and Hispanic callers experienced longer wait times than white patients, indicating a barrier to timely access to primary care.

## Key Points

**Question:**

Is belonging to a racial/ethnic minority group associated with differential access to primary care appointments?

**Findings:**

In this cross-sectional study of 7 simulated black, Hispanic, and white patient callers requesting primary care appointments at 804 randomized physician offices, black and Hispanic callers were asked more frequently about their insurance status. Black and Hispanic callers were offered appointments further in the future despite having the same insurance status as their white counterparts.

**Meaning:**

In this study, black and Hispanic patients faced barriers to timely access to primary care.

## Teaser

This cross-sectional study examines the association between patient race/ethnicity and the rate at which patients are offered primary care appointments and the number of days they wait for their primary care appointments.

## Introduction

On average, individuals who belong to racial and ethnic minority groups experience worse health outcomes than white individuals in the United States.^1,2,3^ While there is a great deal of evidence for the biological and environmental determinants of health disparities, how the health system itself contributes is not as well understood. These disparities may be in part because of discrimination in timely access to services. On average, black and Hispanic patients wait longer than white patients to be seen at their physician’s office^4^ and in the emergency department.^5^ These delays likely contribute to poorer physical and mental health, given that untimely access can exacerbate these conditions and cause anxiety.^6,7^

Many primary care appointments are scheduled over the telephone, when scheduling staff members tend to have discretion over the appointment times that they offer.^8^ Names can signal race, ethnicity, and gender,^9,10,11^ as can voice.^12,13^ Accordingly, appointments scheduled over the telephone likely include racial, ethnic, and gendered signals. It is plausible that biases among scheduling staff result in more patients from minority groups being turned away from an appointment or given later appointments. These disparities could stem from *taste-based discrimination*, defined as instances in which there is animus against minority patients,^14^ or these disparities could result from *statistical discrimination*, defined as instances in which, without information about the individual patient, scheduling staff draw on stereotypes to make assumptions about minority patients’ insurance coverage or ability to pay for services.^15^ Both forms are discriminatory (ie, treating equals unequally^15^), with the former driven by prejudice and the latter driven by imperfect information in pursuit of optimizing practice revenue.

Most related literature links racial and ethnic disparities in health to 1 of 3 determinants: self-reported discrimination,^16,17,18^ explicit bias,^1,2,17,19,20^ or implicit bias toward black and Hispanic patients on the part of health service professionals.^19,21,22,23,24,25^ Explicit bias is typically a bias an individual is aware of and can to some extent control, whereas implicit biases typically occur automatically and are outside of an individual’s awareness and control.^2^ Overall, research suggests that black patients report discrimination from health care professionals significantly more frequently, that health care professionals tend to be significantly more biased toward black and Hispanic patients than white patients, and that implicit bias is more common than explicit bias among health care professionals. While informative, these studies^1,2,16,17,18,19,20,21,22,23,24,25^ generally measure attitudes rather than behaviors, use self-reported measures rather than measured discrimination, and do not control for other factors that may cause the disparities. Field experiments are needed to hold everything constant except for minority status,^26,27,28^ but these studies remain rare in health settings.^29,30^ A few small-scale field experiments have suggested that health care professionals show favoritism based on class,^31,32,33^ insurance status,^30,34,35,36,37,38,39^ race,^30,33,38^ and gender.^30,32,33,38^ Among the field experiments that included race and ethnicity signals,^30,33,38^ the mechanisms by which racial and ethnic discrimination operate remain unclear. For instance, in a 2015 field experiment,^30^ callers attempted to schedule appointments for their aunts and uncles, which required the scheduler to assume the race of the patient based on that of the caller. Furthermore, these callers explicitly signaled insurance status during each call, either by responding to an inquiry about insurance or by asking whether their simulated insurance type was acceptable.^30^ However, the occurrence of an inquiry about insurance could provide insights into what mediates discrimination. Another field experiment among pediatric practices^39^ found that schedulers were 7 times more likely to ask a caller whether their child was a Children’s Health Insurance Program or Medicaid beneficiary if the scheduler thought that the caller was black, suggesting that race may be perceived as associated with insurance status.

In this study, we used a randomized cross-sectional design to examine whether patients from racial and ethnic minority groups experience differential access to medical appointments. We investigated whether there were differences in the rates at which black and Hispanic patients were offered medical appointments and the number of days they waited for their appointments compared with white patients. Importantly, we categorized the types of questions that schedulers asked in the scheduling calling and analyzed the differences in questions asked by race and ethnicity to understand the mechanisms by which discrimination may occur.

## Methods

This study was approved by the Tulane University institutional review board. Callers provided written informed consent. The institutional review board determined that scheduling staff did not constitute human subjects in this research, and therefore, they did not provide informed consent. This study followed Strengthening the Reporting of Observational Studies in Epidemiology (STROBE) reporting guideline.

### Sample Selection

We used the Texas Medical Board database of active licensed physicians to select physician offices. First, we first identified all physicians whose self-reported primary office address was within the study’s geographic area. We then retained primary care physicians and removed duplicate offices based on the address. We defined primary care as being among the following subspecialties: family medicine, family practice, general practice, general preventive medicine, gynecology, internal medicine, obstetrics and gynecology, sleep medicine, endocrinology, and preventive medicine. The resulting list served as the sampling frame of 1888 offices, from which we selected a subset using simple random sampling.

### Data Collectors

We recruited 7 female data collectors (ie, callers). Each caller invented a pseudonym that they felt signaled their gender, racial, and ethnic identity and that they felt comfortable using on the calls. As guidance, we suggested that they pick the first name of a female cousin and the last name of another cousin (eg, Felicia Jackson [black], Emily McConnell [white], Maria Rodriguez [Hispanic]). Callers were prescreened for unusual linguistic backgrounds. In all cases, those recruited had grown up in the United States with biological relatives and reported having lived most of their lives in communities with significant numbers of people whose racial or ethnic identity matched their own.

### Script and Procedures

During each call, the caller started by introducing herself by her pseudonym and asking to be scheduled for the next available appointment as a new patient. Callers did not proactively offer any additional information but did answer any questions that the scheduler asked. All callers used the same procedures and script to supply identical personal information (eMethods 1 and eMethods 2 in the Supplement). If asked about health insurance, the caller explained that she did not have insurance and would pay for the visit herself. If asked to describe the reason for the visit, the callers stated that they had a specific health problem. We chose to report health problems to increase the plausibility of an uninsured patient seeking an appointment and because patients do not typically see some of these specialists if they have no medical issues. We tailored the health problem so that it would be common to the type of practice called but would not constitute a medical emergency (ie, dull stomach pain for general practitioners and gynecologists, difficulty sleeping for sleep medicine specialists, and high blood glucose for endocrinologists).

### Data Collection

Using a standardized data collection form, callers documented information for each primary care practice on their lists. First, they recorded the date and time of the call, whether the call was answered, and the number of minutes that they were on hold, if any. If hold time reached 5 minutes, callers abandoned the attempt. They noted questions or comments made by the schedulers, whether the scheduler had offered them an appointment, and the date and time of the appointment.

### Statistical Analysis

We assessed whether prospective patients were treated differently during the call. To do this, we used the following linear regression analysis and report estimates and statistical significance at the 90%, 95%, and 99% confidence levels using heteroskedasticity-robust SEs: Outcome_i_ = α + β Black_i_ + δ Hispanic_i_ + Controls θ_i_ + Time ϕ_i_ + ε_i_. The estimates on Black_i_ and Hispanic_i_ represent the association of being among the callers in these groups vs white callers. Controls include indicator variables for county (practices were located in 5 counties), county-level variables for socioeconomic, health care market, and demographic characteristics (ie, percentage of residents with a high school diploma or higher, percentage of employed residents, percentage of residents who were born in the United States, percentage of residents who were born in Latin America, density of Hispanic or Latino residents, density black or African American residents, and density Spanish speakers as well as variables for median earnings and whether the areas is classified as a Healthcare Provider Shortage Area, as contained in the randomization check described in the eTable and eAppendix in the Supplement) in addition to indicator variables for practice specialties and physician races. Based on our formal randomization check, we concluded that black callers were no more likely on average to reach practices located in zip codes with different socioeconomic, health care market, and demographic variables than other callers. However, we controlled for these variables in the analyses to account for systematic differences in the locations of practices that Hispanic callers reached, which were in areas with slightly higher educational attainment, higher percentages of individuals with insurance, and a lower density of Hispanic residents. Time included indicator variables for calendar date, day of the week, and a variable for the hour of the call. Hour used whole number values from 8 to 16 (corresponding to 1-hour blocks from 8:00-8:59 am to 4:00-4:59 pm). The times during which callers worked varied depending on their availability.

Because insurance status was the most common question, we evaluated the extent to which including it in the control vector was associated with appointment offers and days to appointment. To assess whether there was discrimination unrelated to concerns about insurance, we conducted the analysis on the subsample in which insurance status was asked and uninsured status was revealed. All estimates were generated using Stata version 14.0 (StataCorp), and statistical significance was set at *P* < .10 using 2-tailed tests.

## Results

### Differential Treatment Leading to an Offer

A total of 7 female simulated patient callers (age range, 18-29 years; 3 [42.9%] non-Hispanic white; 2 [28.6%] non-Hispanic black; 2 [28.6%] bilingual Hispanic) made 1081 calls, which were answered by 804 schedulers from 1888 physician offices in the sampling frame. A total of 42 calls were abandoned when callers were on hold for 5 minutes; excluding these calls did not change the results (data not shown). Overall, 471 (58.6%) calls had complete control data and were included in the analysis. Black callers called a mean of approximately 20 minutes earlier in the day than white callers, while Hispanic callers called approximately 1 hour and 15 minutes later in the day than white callers. Black callers also called a mean of two-thirds of a day later in the week than white callers (eTable, eFigure 1, and eFigure 2 in the Supplement). Black and Hispanic callers were engaged differently in their discussions with practice schedulers (Table 1). While 444 callers (61.2%) were asked about their insurance status, black and Hispanic callers were more likely to be asked than white callers (174 [80.9%] and 183 [63.1%], respectively, vs 114 [38.5%]). The insurance inquiry was the most common question. It was also the question for which the disparities were highest. With controls, black callers were 44.0 (95% CI, 36.2 to 51.8) percentage points more likely to be asked about their insurance status (*P* < .001) and Hispanic callers were 25.3 (95% CI, 17.1 to 33.5) percentage points more likely to be asked about their insurance status (*P* < .001). Caller race or ethnicity was associated with inquiries about insurance status; the discrepancy was not merely a reflection of a different mix of practices located in distinctly different socioeconomic or health care markets (eg, marginal effect of earnings on insurance inquiry in areas with mean income of $31 185, −0.906; 95% CI, −4.101 to 2.289; marginal effect of education level on insurance inquiry in areas with 86.9% of residents with at least a high school diploma, 0; 95% CI, −0.003 to 0.002). (Table 2). Other questions and comments were asked and had statistically significant estimates but occurred at significantly lower frequencies. For example, Hispanic callers were 4.1 (95% CI, 0 to 8.2) percentage points more likely to be asked about marriage status than white callers (*P* = .05) (Table 1).

**Table 1.** Questions Asked by Patient Race and Ethnicity Among 804 Completed Calls

Patient Group	No. (%)						
	Insurance Status	Reason for Visit	Marriage Status	Told Not Accepting New Patients	Ethnicity	Social Security Number	Pregnancy Status
Unadjusted							
White (n = 299)	114 (38.5)	86 (28.7)	11 (3.7)	15 (5.0)	5 (1.7)	7 (2.9)	4 (1.3)
Black (n = 215)	174 (80.9)	30 (13.9)	6 (2.8)	3 (1.4)	1 (0.5)	3 (1.4)	0
Hispanic (n = 290)	183 (63.1)	0	27 (9.3)	4 (1.4)	15 (5.2)	1 (0.1)	0
Marginal effect (95% CI)^a^							
Black	0.440 (0.362 to 0.518)^b^	−0.009 (−0.034 to 0.016)	−0.007 (−0.042 to 0.028)	−0.041 (−0.070 to −0.011)^b^	−0.0126 (−0.032 to 0.007)	−0.150 (−0.223 to −0.077)^b^	−0.015 (−0.029 to 0.000)^c^
Hispanic	0.253 (0.171 to 0.335)^b^	−0.024 (−0.042 to −0.005)^c^	0.041 (0.000 to 0.082)^c^	−0.035 (−0.066 to −0.005)^c^	0.030 (0.000 to 0.061)^d^	−0.295 (−0.349 to −0.241)^b^	−0.014 (−0.027 to 0.000)^c^

^a^ Confidence intervals are calculated using heteroskedasticity-robust SEs. Marginal effects are relative to rates among white patients.

^b^ *P* < .01.

^c^ *P* < .05.

^d^ *P* < .10.

**Table 2.** Association of County-Level Variables With Insurance Inquiry^a^

Variable	Median Earnings	Insured	HPSA	Employed	High School Diploma
Observations, No.	757	768	804	768	768
Mean	$31 185	83.3%	0.75%	68.5%	86.9%
Marginal effect on insurance inquiry (95% CI)^b^	−0.906 (−4.101 to 2.289)	−0.001 (−0.004 to 0.003)	0.080 (−0.298 to 0.458)	−0.001 (−0.005 to 0.004)	0.000 (−0.003 to 0.002)

Abbreviation: HPSA, Healthcare Provider Shortage Area.

^a^ These estimates were run without controls because they are part of the control vector. Observations vary because data were not evenly available for every zip code.

^b^ Confidence intervals are calculated using heteroskedasticity-robust SEs.

### Differential Treatment in Offer and Days to Offer

Overall, 582 callers (72.4%) were offered appointments. In the unadjusted models, black and Hispanic callers were more likely to be offered an appointment compared with white callers (black callers, 32.2 [95% CI, 25.1 to 39.3] percentage points more likely; *P* < .001; Hispanic callers, 21.1 [95% CI, 13.7 to 28.5] percentage points more likely; *P* < .001) (Table 3). The addition of controls did not change the results for offer rates, suggesting that differences in outcomes by caller race or ethnicity were not associated with potential differences in the cohorts of practices that they called. The addition of time did not change the results for offer rates, but controlling for whether insurance status was asked caused all estimates on black and Hispanic callers receiving appointment offers to lose statistical significance (black callers, 4.7 [95% CI, −4.7 to 14.2] percentage points; *P* = .33; Hispanic callers, 4.9 [95% CI, −3.3 to 13.2] percentage points; *P* = .24). Callers who were asked about insurance status were 50.7 (44.0 to 57.4) percentage points more likely to be offered an appointment (*P* < .001), which suggests that significantly more appointments were offered if insurance status was revealed.

**Table 3.** Offer Rates by Patient Race and Ethnicity

Patient Group	Unadjusted	Controls^a^	Time^b^	Controls and Time	Controls, Time, and Subsample^c^
Observations, No.	804	757	804	757	443
Offered an appointment, No. (%)					
White	168 (56.2)	NA	NA	NA	NA
Black	190 (88.4)	NA	NA	NA	NA
Hispanic	224 (77.2)	NA	NA	NA	NA
Marginal effect (95% CI)^d^					
Black	0.322 (0.251 to 0.393)^e^	0.322 (0.249 to 0.395)^e^	0.224 (0.115 to 0.333)^e^	0.047 (−0.047 to 0.142)	0.052 (−0.013 to 0.117)
Hispanic	0.211 (0.137 to 0.285)^e^	0.207 (0.129 to 0.285)^e^	0.196 (0.107 to 0.285)^e^	0.0492 (−0.033 to 0.132)	0.002 (−0.076 to 0.080)
Insurance asked	NA	NA	NA	0.507 (0.440 to 0.574)^e^	NA

Abbreviation: NA, not applicable.

^a^ Controls include indicator variables for county, county-level variables for socioeconomic, health care market, and demographic characteristics (ie, percentage of residents with a high school diploma or higher, percentage of employed residents, percentage of residents who were born in the United States, percentage of residents who were born in Latin America, density of Hispanic or Latino residents, density black or African American residents, and density Spanish speakers as well as variables for median earnings and whether the areas is classified as a Healthcare Provider Shortage Area) and indicator variables for practice specialties and physician races. Observations vary when adding controls since American Community Survey data are not available for every zip code for all variables.

^b^ Time includes indicator variables for calendar date, day of the week, and hour of the call.

^c^ Subsample consists of callers asked about their insurance status.

^d^ Confidence intervals are calculated using heteroskedasticity-robust SEs. Marginal effects are relative to rates among white patients.

^e^ *P* < .01.

Among the 582 callers offered appointments, the mean time until that appointment was 10.8 calendar days (SE, 0.6 days; 95% CI, 9.6-12.0 days). In the unadjusted model, the marginal effect estimates for black and Hispanic callers were statistically significant, indicating that black and Hispanic callers received later appointments compared with white callers (black callers: marginal effect estimate, 3.650; 95% CI, 0.579 to 6.721; *P* = .08; Hispanic callers: marginal effect estimate, 2.644; 95% CI, −0.496 to 5.784; *P* = .02) (Table 4 and Figure). The results were unaffected by all controls except time, which caused the effects to become imprecise and statistically insignificant. Among Hispanic callers, who called a mean of approximately 1 hour and 15 minutes later in the day than white callers, the point estimates become much smaller (0.24 [95% CI, −3.70 to 4.18] days later than white callers; *P* = .89). In the final regression model, the estimate for the hour of the day when the call was placed was associated with the days to appointment, indicating that calls later in the day led to later appointments (marginal effect, 1.22 days; 95% CI, 0.44 to 2.00 days; *P* = .002). When restricting the analysis to those who were asked about insurance, black callers were offered appointments a mean of 7.04 (95% CI, 1.78 to 12.31) days later than white callers (*P* = .009).

**Table 4.** Days to Appointment by Patient Race and Ethnicity

Patient Group	Unadjusted	Controls^a^	Time^b^	Controls and Time	Controls, Time, and Subsample^c^
Observations, No.	581	548	581	548	420
Time to appointment, mean (95% CI), d					
White	8.6 (6.1-11.0)	NA	NA	NA	NA
Black	12.2 (10.4-14.1)	NA	NA	NA	NA
Hispanic	11.2 (9.2-13.2)	NA	NA	NA	NA
Marginal effect (95% CI)^d^					
Black	3.650 (0.579 to 6.721)^e^	4.225 (1.175 to 7.275)^f^	3.464 (−0.971 to 7.899)	4.128 (−0.539 to 8.795)^g^	7.046 (1.781 to 12.311)^f^
Hispanic	2.644 (−0.496 to 5.784)^g^	3.660 (0.526 to 6.794)^e^	0.243 (−3.697 to 4.183)	1.253 (−2.634 to 5.140)	3.532 (−0.404 to 7.468)^g^
Insurance asked	NA	NA	NA	−1.323 (−5.127 to 2.481)	NA

Abbreviation: NA, not applicable.

^a^ Controls include indicator variables for county, county-level variables for socioeconomic, health care market, and demographic characteristics (ie, percentage of residents with a high school diploma or higher, percentage of employed residents, percentage of residents who were born in the United States, percentage of residents who were born in Latin America, density of Hispanic or Latino residents, density black or African American residents, and density Spanish speakers as well as variables for median earnings and whether the area is classified as a Healthcare Provider Shortage Area) and indicator variables for practice specialties and physician races. Observations vary when adding controls since American Community Survey data are not available for every zip code for all variables.

^b^ Time includes indicator variables for calendar date, day of the week, and hour of the call.

^c^ Subsample consists of callers asked about their insurance status.

^d^ Confidence intervals are calculated using heteroskedasticity-robust SEs. Marginal effects are relative to white callers.

^e^ *P* < .05.

^f^ *P* < .01.

^g^ *P* < .10.

**Figure.**
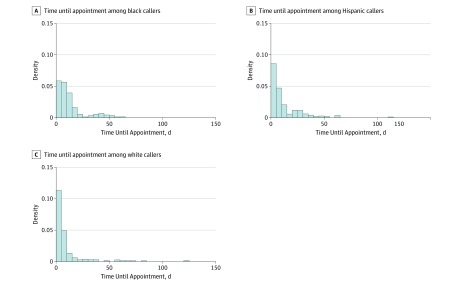
Distribution of Wait Days by Race and Ethnicity

## Discussion

In this study, black and Hispanic callers seeking medical appointments were treated differently than white callers. Overall, schedulers offered appointments to black and Hispanic callers at higher rates than white patients, meaning that we did not find evidence of discrimination against minority patients in terms of access to appointments. However, among those who were offered an appointment, black and Hispanic patients waited longer on average for the appointment, indicating that they may face barriers to timely access to appointments. Black and Hispanic callers were also asked about their insurance status significantly more often, and those asked were offered appointments at different rates. Schedulers may have believed that race and ethnicity were associated with insurance status, and those who asked about insurance appeared to be inquiring in response to race and ethnicity signals because race and insurance status were associated after controlling for other factors. This inquiry would constitute statistical discrimination. These offer rate results may also be a consequence of our design, given that all callers were uninsured, which was only revealed when asked. Black and Hispanic callers whose insurance status was disclosed and who were offered appointments tended to be given appointments further in the future than white callers, who disclosed identical insurance status when asked. Asking about insurance may imply scheduling staff’s concern about the caller’s ability to pay. If practices that did not ask about insurance status assumed that black and Hispanic patients were more likely to be insured by Medicaid, they might have offered these patients appointments less frequently.

Our finding that disclosing uninsured status was positively associated with an appointment offer is surprising because we presumed that schedulers would have a bias against the uninsured. However, scheduling staff appeared to be distinguishing patient groups by Medicaid vs non-Medicaid status rather than insured vs uninsured status. Texas Medicaid disproportionately covers individuals who belong to minority racial/ethnic groups. Although white residents constitute 42% of the Texas population, they represent only 21% of the Medicaid population.^40,41^ A 2014 field experiment in Texas^37^ found that uninsured and private insurance patients were treated similarly, while those with Medicaid were offered fewer appointments. However, determining whether scheduling staff in Texas disproportionately ask minority patients about their insurance status because of a higher expectation of Medicaid was beyond the scope of this study.

Our finding that black and Hispanic patients waited significantly longer for appointments than white patients was not sensitive to an inquiry regarding insurance. This suggests that these results are unlikely to be associated with insurance status. We were unable to ascribe these results to animus or other forms of statistical discrimination, such as assumptions that white callers are less willing to accept long wait times, that minority callers require more time to prepare for the visit, or that there will be systematic differences in the ability to pay out-of-pocket fees. Whether these or other forms of statistical or taste-based discrimination were at play is an important question but beyond the scope of this study.

This study should be replicated using a nationally representative sample of primary care offices and expanded to include callers with a broader range of gender identities, ages, and illness severities. While we found that being asked more often about insurance increased appointment offers for black and Hispanic patients in Texas, had their insurance type been Medicaid or had the market preferences for insurance been different, it is possible that the association of the insurance inquiry with an appointment offer would have been negative. This result needs to be further explored by randomizing insurance status. This would allow for findings to be tested at the national level and would provide more information on the mechanisms of discrimination.

### Limitations

This study has several limitations in scope. Our data collectors were all women with similar ages and education levels who self-identified as members of only 3 racial/ethnic groups. The study was limited to primary care providers, which included endocrinologists and sleep medicine specialists, in urban settings in Texas. Furthermore, we only sought to schedule appointments by telephone, excluding other means by which appointments can be scheduled, such as the internet. We did not quantify the degree to which race and ethnicity were signaled by our data collectors through their assumed names and voices.

## Conclusions

The results of this study suggest that black and Hispanic patients experienced barriers to timely access to primary care when faced with a human gatekeeper to schedule an appointment. Technological options, such as automated scheduling systems and online portals, may mitigate the associations found in this study, particularly for routine services that do not require triage. Solutions that remove the human gatekeeper to the next available appointment might be useful in reducing disparities in access if they do not create new barriers that disproportionately affect minority groups. Additionally, the routine collection and use of data related to disparities in access at both the system and the individual practice level may motivate behavioral and operational change within health care organizations. Future research should investigate whether these systems achieve better equity in access.

## Notes

Supplement. **eMethods 1. **Data Collection Procedures **eMethods 2. **Call Script **eAppendix. **Randomization Check **eTable. **Verification of Randomization **eFigure 1.** Calls by Day of the Week and Caller Race/Ethnicity **eFigure 2.** Calls by Hour of the Day and Caller Race/Ethnicity

## References

[1] Blair IV, Havranek EP, Price DW, Assessment of biases against Latinos and African Americans among primary care providers and community members. Am J Public Health. 2013;103(1):-. doi:10.2105/AJPH.2012.300812 23153155 PMC3518332

[2] Dovidio JF, Kawakami K, Gaertner SL Implicit and explicit prejudice and interracial interaction. J Pers Soc Psychol. 2002;82(1):62-68. doi:10.1037/0022-3514.82.1.62 11811635

[3] Horn IB, Mendoza FS Reframing the disparities agenda: a time to rethink, a time to focus. Acad Pediatr. 2014;14(2):115-116. doi:10.1016/j.acap.2013.12.005 24602572

[4] Ray KN, Chari AV, Engberg J, Bertolet M, Mehrotra A Disparities in time spent seeking medical care in the United States. JAMA Intern Med. 2015;175(12):1983-1986. doi:10.1001/jamainternmed.2015.4468 26437386 PMC5055855

[5] James CA, Bourgeois FT, Shannon MW Association of race/ethnicity with emergency department wait times. Pediatrics. 2005;115(3):e310-e315. doi:10.1542/peds.2004-1541 15741357

[6] Boudreau RM, McNally C, Rensing EM, Campbell MK Improving the timeliness of written patient notification of mammography results by mammography centers. Breast J. 2004;10(1):10-19. doi:10.1111/j.1524-4741.2004.09608.x 14717754

[7] Himelhoch S, Weller WE, Wu AW, Anderson GF, Cooper LA Chronic medical illness, depression, and use of acute medical services among Medicare beneficiaries. Med Care. 2004;42(6):512-521. doi:10.1097/01.mlr.0000127998.89246.ef 15167319

[8] Brandenberg L, Grabow P, Steel G, Toussaint J, Tyson B Innovation and best practices in health care scheduling. https://nam.edu/wp-content/uploads/2015/06/SchedulingBestPractices.pdf. Accessed December 12, 2019.

[9] Barlow MR, Lahey JN What race is Lacey? intersecting perceptions of racial minority status and social class. Soc Sci Q. 2018;99(5):1680-1698. doi:10.1111/ssqu.12529

[10] Fryer RG, Levitt SD The causes and consequences of distinctly black names. Q J Econ. 2004;119(3):767-805. doi:10.1162/0033553041502180

[11] Gaddis SM Racial/ethnic perceptions from Hispanic names: selecting names to test for discrimination. Socius. 2017;3:1-11. doi:10.1177/2378023117737193

[12] Baugh J Linguistic profiling In: Makoni S, Smitherman G, Ball AF, Spears AK, eds. Black Linguistics: Language, Society, and Politics in Africa and the Americas. New York, NY: Routledge; 2003:155-168.

[13] Chin WY Linguistic profiling in education: how accent bias denies equal educational opportunities to students of color. Scholar. 2010;12:355-384. http://lawspace.stmarytx.edu/item/STMU_TheScholarStMarysLRev_v12i3p0355_Chin. Accessed December 12, 2019.

[14] Becker G The Economics of Discrimination. Chicago, IL: Chicago University Press; 1957.

[15] Phelps E Inflation Policy and Unemployment Theory: The Cost-Benefit Approach to Monetary Planning. London, United Kingdom: Macmillan Publishers; 1972.

[16] Hausmann LR, Jeong K, Bost JE, Ibrahim SA Perceived discrimination in health care and health status in a racially diverse sample. Med Care. 2008;46(9):905-914. doi:10.1097/MLR.0b013e3181792562 18725844 PMC3424509

[17] Penner LA, Dovidio JF, West TV, Aversive racism and medical interactions with black patients: a field study. J Exp Soc Psychol. 2010;46(2):436-440. doi:10.1016/j.jesp.2009.11.004 20228874 PMC2835170

[18] Ryan AM, Gee GC, Griffith D The effects of perceived discrimination on diabetes management. J Health Care Poor Underserved. 2008;19(1):149-163. doi:10.1353/hpu.2008.0005 18263991

[19] Sabin J, Nosek BA, Greenwald A, Rivara FP Physicians’ implicit and explicit attitudes about race by MD race, ethnicity, and gender. J Health Care Poor Underserved. 2009;20(3):896-913. doi:10.1353/hpu.0.0185 19648715 PMC3320738

[20] Sabin JA, Rivara FP, Greenwald AG Physician implicit attitudes and stereotypes about race and quality of medical care. Med Care. 2008;46(7):678-685. doi:10.1097/MLR.0b013e3181653d58 18580386

[21] Chapman EN, Kaatz A, Carnes M Physicians and implicit bias: how doctors may unwittingly perpetuate health care disparities. J Gen Intern Med. 2013;28(11):1504-1510. doi:10.1007/s11606-013-2441-1 23576243 PMC3797360

[22] FitzGerald C, Hurst S Implicit bias in healthcare professionals: a systematic review. BMC Med Ethics. 2017;18(1):19. 28249596 10.1186/s12910-017-0179-8 PMC5333436

[23] Hall WJ, Chapman MV, Lee KM, Implicit racial/ethnic bias among health care professionals and its influence on health care outcomes: a systematic review. Am J Public Health. 2015;105(12):e60-e76. doi:10.2105/AJPH.2015.302903 26469668 PMC4638275

[24] Krieger N, Carney D, Lancaster K, Waterman PD, Kosheleva A, Banaji M Combining explicit and implicit measures of racial discrimination in health research. Am J Public Health. 2010;100(8):1485-1492. doi:10.2105/AJPH.2009.159517 19965567 PMC2901297

[25] Maina IW, Belton TD, Ginzberg S, Singh A, Johnson TJ A decade of studying implicit racial/ethnic bias in healthcare providers using the implicit association test. Soc Sci Med. 2018;199:219-229. doi:10.1016/j.socscimed.2017.05.009 28532892

[26] Neumark D Experimental research on labor market discrimination. J Econ Lit. 2018;56(3):799-896. doi:10.1257/jel.20161309

[27] Bertrand M, Duflo E Field experiments on discrimination. https://economics.mit.edu/files/11449. Accessed December 12, 2019.

[28] Gaddis SM Discrimination in the credential society: an audit study of race and college selectivity in the labor market. Soc Forces. 2015;93(4):1451-1479. doi:10.1093/sf/sou111

[29] Hansen F The future of health economics: the potential of behavioral and experimental economics. Nordic J Health Econ. 2015;3(1):68-86. doi:10.5617/njhe.660

[30] Sharma R, Mitra A, Stano M Insurance, race/ethnicity, and sex in the search for a new physician. Econ Lett. 2015;137:150-153. doi:10.1016/j.econlet.2015.11.005

[31] Angerer S, Waibel C, Stummer H Discrimination in health care: a field experiment on the impact of patients’ socio-economic status on access to care. https://papers.ssrn.com/sol3/papers.cfm?abstract_id=3036000. Accessed December 12, 2019.

[32] Olah ME, Gaisano G, Hwang SW The effect of socioeconomic status on access to primary care: an audit study. CMAJ. 2013;185(6):E263-E269. doi:10.1503/cmaj.121383 23439620 PMC3612171

[33] Kugelmass H “Sorry, I’m not accepting new patients”: an audit study of access to mental health care. J Health Soc Behav. 2016;57(2):168-183. doi:10.1177/0022146516647098 27251890

[34] Bisgaier J, Rhodes KV Auditing access to specialty care for children with public insurance. N Engl J Med. 2011;364(24):2324-2333. doi:10.1056/NEJMsa1013285 21675891

[35] Olin SC, O’Connor BC, Storfer-Isser A, Access to care for youth in a state mental health system: a simulated patient approach. J Am Acad Child Adolesc Psychiatry. 2016;55(5):392-399. doi:10.1016/j.jaac.2016.02.014 27126853 PMC4970515

[36] Polsky D, Richards M, Basseyn S, Appointment availability after increases in Medicaid payments for primary care. N Engl J Med. 2015;372(6):537-545. doi:10.1056/NEJMsa1413299 25607243

[37] Rhodes KV, Kenney GM, Friedman AB, Primary care access for new patients on the eve of health care reform. JAMA Intern Med. 2014;174(6):861-869. doi:10.1001/jamainternmed.2014.20 24710808

[38] Sharma R, Tinkler S, Mitra A, Pal S, Susu-Mago R, Stano M State Medicaid fees and access to primary care physicians. Health Econ. 2018;27(3):629-636. doi:10.1002/hec.3591 28944526

[39] Leech TGJ, Irby-Shasanmi A, Mitchell AL “Are you accepting new patients?” a pilot field experiment on telephone-based gatekeeping and Black patients’ access to pediatric care. Health Serv Res. 2019;54(suppl 1):234-242. doi:10.1111/1475-6773.13089 30506767 PMC6341201

[40] Claxton G, Rae M, Long M, Damico A, Whitmore H Employer health benefits 2018 annual survey. https://www.kff.org/health-costs/report/2018-employer-health-benefits-survey/. Accessed December 12, 2009.

[41] United States Census Bureau Community facts. https://factfinder.census.gov/faces/nav/jsf/pages/community_facts.xhtml. Accessed June 16, 2019.

