# Supplementary material for: Association of Simulated Patient Race/Ethnicity With Scheduling of Primary Care Appointments
Source: JAMA Netw Open. 2020 Jan 29;3(1):e1920010. doi: 10.1001/jamanetworkopen.2019.20010 (PMC6991290; doi:10.1001/jamanetworkopen.2019.20010)
Supplement: Supplement. — eMethods 1. Data Collection Procedures eMethods 2. Call Script eAppendix. Randomization Check eTable. Verification of Randomization eFigure 1. Calls by Day of the Week and Caller Race/Ethnicity eFigure 2. Calls by Hour of the Day and Caller Race/Ethnicity [file jamanetwopen-3-e1920010-s001.pdf]

# Supplementary Online Content

Wisniewski JM, Walker B. Association of simulated patient race/ethnicity with scheduling of primary care appointments. *JAMA Netw Open*. 2020;3(1):e1920010. doi:10.1001/jamanetworkopen.2019.20010

**eMethods 1.** Data Collection Procedures
**eMethods 2.** Call Script
**eAppendix.** Randomization Check
**eTable.** Verification of Randomization
**eFigure 1.** Calls by Day of the Week and Caller Race/Ethnicity
**eFigure 2.** Calls by Hour of the Day and Caller Race/Ethnicity

This supplementary material has been provided by the authors to give readers additional information about their work.

© 2020 Wisniewski JM et al. *JAMA Network Open.*

## eMethods1. Data Collection Procedures

### Audit Procedure Instructions Provided to Research Assistants

- Call the practice using a Texas Google voice number.
- As soon as an appointment date/time is offered, say that you have to check your work schedule to see whether you can get that day off, and that you'll call back. Do not schedule an appointment.
- **No answer:** If there is no answer, call back up to two more times during the scheduled calling session, then give up.
- **Leave a voicemail:** Do not leave a voicemail. Call back up to two more times during the scheduled calling session, then give up.
- **On hold:** If put on hold for more than five minutes, hang up and do not call back.
- **Online scheduling:** If you referred to an online appointment system, contact the study team.
- **Charity clinic:** If they recommend that you go to a charity clinic, thank them and record that no appointment was offered.
- **Call backs:** If the scheduler offers to call you back, give them the telephone number.
- **Out of time:** If you cannot finish your calls during the scheduled calling session, or if you have to miss a session, contact the study team as soon as possible.

### Call Logistics

Data collection occurred between November 2017 and February 2018. We assigned each caller a unique list of randomly selected practices to call. Callers used physician names and addresses to find telephone numbers through internet searches. In the case that they could not find a telephone number, that practice was skipped.[1] If there was no answer at an office, callers tried again during the same two-hour period, then abandoned the attempt. We called offices once. Callers used telephone numbers with area codes that corresponded to the city that they were calling. They made all calls during regular business hours. If the scheduler placed the caller on hold, she remained on hold up to five minutes and then hung up. Callers did not leave a message if asked. Calls referred to an online scheduling system were coded as "no appointment offered,"[2] as were referrals to other practices such as charity clinics.[3] We instructed the Hispanic callers to conduct the call in Spanish if offered.[4] All other calls were conducted in English.

---

[1] In total, we skipped 263 practices because we could not find a phone number.
[2] Only four instances of referral to an online system were logged.
[3] Only two cases of a referral were recorded.
[4] We recorded only twelve instances of an option to speak Spanish.

© 2020 Wisniewski JM et al. *JAMA Network Open.*

## eMethods2. Call Script

| Scheduler Question | Secret Shopper Response |
|---|---|
| Are you a new patient? / Is this your first time seeing the doctor? | Yes |
| When would you like to come in? / Do you prefer morning or afternoon? | What is the next appointment you have open?<br>How soon can I get in? (If they ask whether you can come today, ask them what time today, then say no.) |
| What are you coming in for? / Why do you want to see the doctor? | If calling a GP or Ob Gyn: I'm having a lot of pain in my lower stomach. It's been about two weeks. Dull pain, comes and goes (convince them it's not an emergency.)<br><br>If calling a sleep med doctor: I'm having a lot of trouble sleeping. Only sleeping 2-3 hours per night. It's been about two weeks.<br><br>If calling an endocrinologist: I had my blood sugar tested (at a health fair) and it was high. They told me to make an appointment with a doctor. |
| When was the last time you saw a doctor, had a routine exam, etc.? | About three years ago (in San Antonio) |
| Could you be pregnant? Are you using birth control? | I don't think so. Yes, condoms. |
| Home address | (Same for each city) |
| Insurance company | I don't have insurance. I'll be paying for it. |
| Do you understand that you will be responsible for the cost of your visit? | Yes, that's OK. |
| Social security number | I don't know it. Let me find my card and call you back. |
| Phone number | Google voice number (each secret shopper will have own). |
| Date of birth / How old are you? | October 23, 1993; You're 24 years old. |
| Marital status | Single |
| Occupation | Work at Kroger (cashier at a grocery store) |
| Our practice has multiple locations. Can you go to a different one? | Yes |
| Which doctor do you want to see? | It doesn't matter. No preference |
| Do you prefer a male or female doctor? | No, whoever has the earliest opening is fine. |

© 2020 Wisniewski JM et al. *JAMA Network Open.*

## eAppendix. Randomization Check

We first assessed whether our randomization worked. To do this, we regressed various socioeconomic, market, and demographic variables by ZIP Code (sourced from American FactFinder) against race (e.g., Black) and ethnicity (e.g., Hispanic) indicator variables to determine whether Black and Hispanic callers had a systematically different likelihood of reaching practices in a different environment. We specifically assessed variables for socioeconomic factors (e.g., percent high school graduate or higher, percent employed, and median earnings), market factors (e.g., percent insured and whether the area constituted a health care provider shortage area), and race, ethnicity, and nativity variables (e.g., percent born in the United States, percent born in Latin America, percent Hispanic or Latino, percent Black or African American, and percent Spanish speakers) (US Census American FactFinder, 2018). We used the following linear probability model specification:

$$Factor_i = \alpha + \beta\ Black_i + \delta\ Hispanic_i + \varepsilon_i, \quad [2]$$

Where $i$ indicates each call, $Black_i$ is an indicator variable for Black callers, $Hispanic_i$ is an indicator variable for Hispanic callers, and the omitted group is White callers. We computed confidence intervals using heteroskedasticity-robust standard errors.

The randomization appears to have generally worked. Of the ten variables tested that were related to the practice locations, only one had a statistically significant estimate on an indicator for Black callers (employment rate at the 90% confidence level; see Table 1). We expect some statistically significant results due to chance, and one of ten estimates will be statistically significant at the 10% level on average with no actual effect (which, incidentally, is what we find). Three variables had a statistically significant estimate on an indicator for Hispanic callers (percent high school graduation rate at the 95% confidence level, percent insured at 90% confidence level and percent Hispanic or Latino at the 90% confidence level). Hispanic callers reached practices in areas that were slightly more educated, more insured, and less Hispanic. Consequently, we conclude that Black callers were no more likely on average to reach practices located in ZIP Codes with different socioeconomic, market, and demographic variables. However, we control for these variables in the analyses to account for systematic differences in the locations of practices that Hispanic callers reached (the results are largely unchanged for the estimates on Black or Hispanic callers, as can be seen in Table 2 for offer rates and Table 3 for days-to-appointment).

© 2020 Wisniewski JM et al. *JAMA Network Open.*

## eTable. Verification of Randomization

| | | | | |
|---|---|---|---|---|
| Randomization Outcome | White Mean | N | Black-White Difference [95% CI] | Hispanic-White Difference [95% CI] |
| *Socioeconomic and Market Variables* | | | | |
| Percent High School Graduate | 86.3% | 768 | 1.71% [-0.56%, 3.98%] | 2.57%** [0.57%, 4.57%] |
| Percent Employed | 67.4% | 768 | 1.38%* [-0.07%, 2.83%] | 0.74% [-0.44%, 1.91%] |
| Median Earnings | $31,565 | 757 | $224 [-$1,828, $2,277] | $156 [-$1,688, $2,000] |
| Percent Insured | 83.3% | 768 | 0.60% [-1.33%, 2.53%] | 1.53%* [-0.16%, 3.22%] |
| Healthcare Provider Shortage Area | 1.00% | 804 | -0.07% [-0.02%, 0.02%] | -0.01% [-0.02%, 0.01%] |
| *Race, Ethnicity, and Nativity Variables* | | | | |
| Percent Born in US | 76.6% | 768 | -1.21% [-3.15%, 0.73%] | 0.84% [-0.85%, 2.53%] |
| Percent Born in Latin America | 48.5% | 768 | -3.76% [-8.52%, 1.00%] | -2.12% [-6.38%, 2.13%] |
| Percent Hispanic or Latino | 26.7% | 768 | -1.46% [-4.99%, 2.07%] | -2.90%* [-5.88%, 0.08%] |
| Percent Black or African American | 15.4% | 768 | -1.38% [-3.86%, 1.10%] | -0.09 [-2.46, 2.28] |
| Percent Spanish Speakers | 21.8% | 768 | -0.99% [-4.29%, 2.32%] | -2.45% [-5.25%, 0.35%] |
| *Practice Characteristics* | | | | |
| Family Medicine Specialist | 39.5% | 804 | 0.05 [-0.04, 0.13] | 0.01 [-0.07, 0.09] |
| General Medicine Specialist | 5.4% | 804 | -0.03 [-0.06, 0.01] | 0.01 [-0.3, 0.05] |
| Internal Medicine Specialist | 34.8% | 804 | -0.04 [-0.12, 0.04] | -0.02 [-0.10, 0.05] |
| OB/GYN Specialist | 18.7% | 804 | 0.01 [-0.06, 0.08] | -0.00 [-0.07, 0.06] |
| *Call Characteristics* | | | | |
| Day of Week (see notes) | 3.5 | 804 | 0.64*** [0.45, 0.83] | -0.06 [-0.24, 0.11] |
| Hour of Day (see notes) | 11.9 | 804 | -0.28* [-0.60, 0.03] | 1.21 [0.94, 1.48] |
| Minutes on Hold | 0.74 | 804 | -0.50*** [-0.71, -0.29] | 0.01 [-0.22, 0.24] |
| Call Answered | 0.67 | 1,081 | 0.17*** [0.10, 0.23] | 0.09*** [0.03, 0.15] |
| | | | | |

Notes: Different from zero at 1-percent level (***), 5-percent level (**) or 10-percent level (*). Confidence intervals are calculated using heteroskedasticity-robust standard errors. *Day of Week* is coded as Monday =1, …, Friday=5. *Hour of Day* has a range of 8-16 corresponding with 8am-4pm**.**

**eFigure 1.** Calls by Day of the Week and Caller Race/Ethnicity

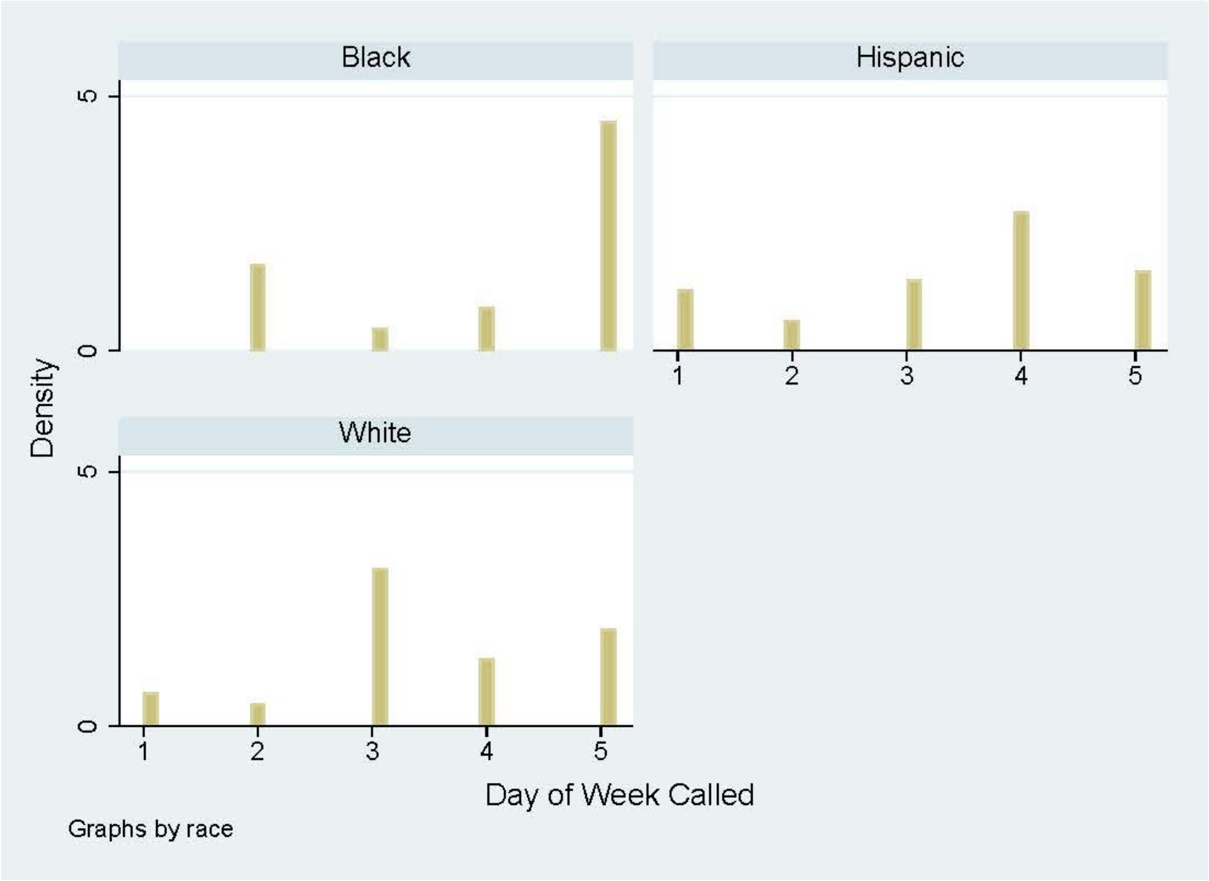


© 2020 Wisniewski JM et al. *JAMA Network Open.*

**eFigure 2.** Calls by Hour of the Day and Caller Race/Ethnicity

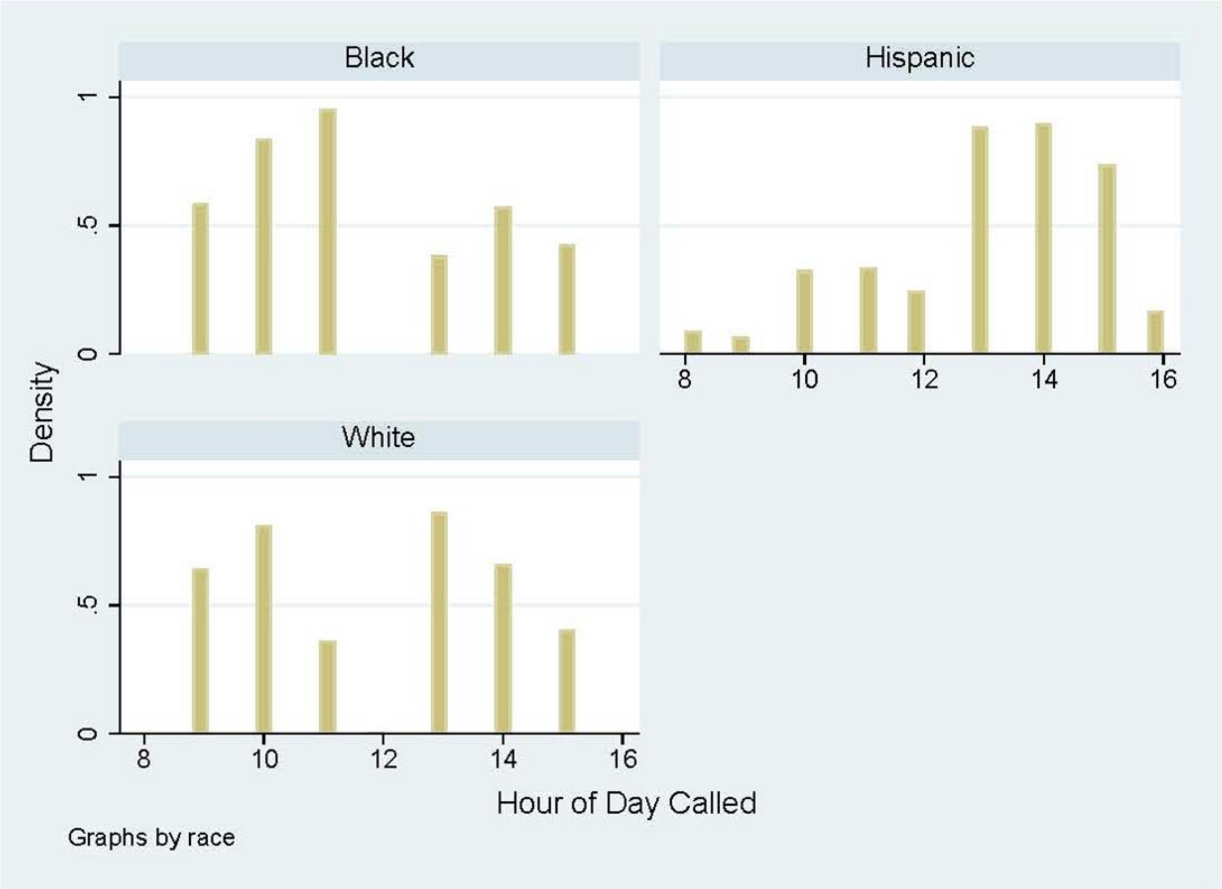


© 2020 Wisniewski JM et al. *JAMA Network Open.*
